# Paradigms of pathogenesis: targeting the mobile genetic elements of disease

**DOI:** 10.3389/fcimb.2012.00161

**Published:** 2012-12-14

**Authors:** Eric C. Keen

**Affiliations:** Department of Biology, University of MiamiCoral Gables, FL, USA

A century ago, Gertrude Stein told us that a rose is a rose is a rose, but today, modern genomics is telling us that a pathogen is not a pathogen. Advances in pathogenomics have greatly elucidated the genetic and molecular underpinnings of bacterial virulence and pathogenesis. This new knowledge of the evolution of pathogenic bacteria, and of the ways by which they acquire and maintain virulence, has increasingly indicated that not all bacterial pathogens are created equal. Evolutionarily speaking, there seem to be at least two broad categories of pathogenic bacteria: obligate pathogens that have evolved over time to become irreversibly specialized parasites and “Jekyll-and-Hyde pathogens,” still closely related to free-living bacteria, that have been rapidly but reversibly made pathogenic by mobile genetic elements. This distinction between full-scale genetic re-wiring and subtle genetic fine-tuning represents a fundamental contrast that may shed light on the past, present, and future evolution of pathogenic bacteria. More importantly, we might be able to use this knowledge of the paradigms of pathogenesis to develop novel strategies for combating some of today's most significant bacterial pathogens.

With the tremendous amount of information that has been generated by the genomics revolution, it is not surprising that new paradigms for the evolution of bacterial pathogens have emerged. One such paradigm is that bacteria can be pathogenic not because they possess defined “virulence factors” but because they have deleted genes detrimental to a pathogenic lifestyle (Maurelli et al., 1998) and/or have lost metabolic and regulatory genes required for a free-living state (Georgiades and Raoult, 2011). This principle of reductive evolution, which posits that certain bacteria gain virulence via massive gene deletion associated with increased specialization, has been most extensively documented amongst obligate intracellular pathogens and seems to represent a dominant evolutionary phenomenon of particularly specialized bacteria (Merhej et al., 2009). Nevertheless, massive gene loss is only a part of a larger picture for many other pathogenic bacteria. In fact, certain bacterial pathogens can be readily distinguished from non-pathogenic, free-living kin not by massive gene deletion but by specific virulence factors. Since non-pathogenic bacteria can chromosomally encode certain structural virulence factors previously considered unique to pathogens, such as type III and type VI secretion systems (Pallen and Wren, 2007), it is important to emphasize that the pathogenicity of these Jekyll-and-Hyde bacteria is largely mediated by mobile genetic elements such as bacteriophages and plasmids.

The most prominent examples of these Jekyll-and-Hyde bacteria are those whose virulence factors, typically exotoxins and/or extracellular enzymes, are encoded by temperate bacteriophages. Free-living or commensal bacteria that pose little threat to human health can be rapidly transformed by lysogenic conversion into aggressively virulent pathogens. For example, uninfected strains of *Clostridium botulinum*, *Corynebacterium diphtheriae*, *Escherichia coli*, *Staphylococcus aureus*, *Streptococcus pyogenes*, and *Vibrio cholerae* are not usually dangerous, but those lysogens that acquire just a few exogenous genes from one or several virulence factor-encoding bacteriophage(s) can become highly virulent (Brussow et al., 2004). In this light, one could even make the case that the cholera toxin-encoding bacteriophage CTXϕ, not the bacterium *V. cholerae*, is the true causative agent of the disease we know as cholera. Similarly, a comparative genomic analysis of three taxonomic species within the genus *Bacillus*—*B. anthracis*, *B. cereus*, and *B. thuringiensis—*suggests that these three bacteria with vastly different ecological niches (acute pathogen, ubiquitous soil dweller, and insect commensal, respectively) are genetically a single species (Helgason et al., 2000). The genomes of these three bacteria are of similar sizes, and massive gene deletion does not explain *B. anthracis*'s extreme virulence compared to its kin; instead, its pathogenicity is largely a product of certain virulence factors encoded by two plasmids, without which anthrax is not fully virulent (Helgason et al., 2000). In all of these cases, the presence or absence of one or more virulence factor-encoding prophages and/or plasmids strongly influences their carriers' virulence and pathogenicity.

Of course, not all pathogenic bacteria can be neatly categorized into two camps: some pathogens, like *Shigella* spp., differ from their free-living kin both by gene deletion and by extrachromosomal virulence factors (Johnson, 2000). Indeed, these two paradigms of pathogenesis—gene deletion and the acquisition of mobile genetic elements—are not necessarily mutually exclusive because they operate over different scales of evolutionary time. In contrast to the classical evolution of bacteria, which occurs over many thousands and millions of years, horizontal gene transfer represents the fast mode of evolution, one by which prokaryotes can acquire new genes conferring unique traits, including virulence, in a very short time (Brussow et al., 2004). There are also undoubtedly other dynamics, such as epigenetics, that play important roles in the evolution and maintenance of virulence (Casadesus and Low, 2006). Nevertheless, it is important to recognize that the pathogenic bacteria confronting us today have, in many cases, gained pathogenicity through fundamentally different mechanisms. Despite their obvious differences, *V. cholerae* and *B. anthracis* are both examples of a larger evolutionary phenomenon: rather than becoming pathogenic by losing the genes whose loss would irreversibly separate them from their free-living relatives, as do obligate intracellular pathogens, Jekyll-and-Hyde bacteria differ from their free-living relatives, and are made pathogenic, primarily by their mobile genetic elements.

What practical value does this distinction have? The fact that bacterial virulence can sometimes be directly attributed to mobile genetic elements has potentially significant biomedical implications. If we refine our focus to implicate those mobile genetic elements as the underlying causes of the diseases traditionally attributed to their bacterial hosts, we open the door to the possibility of novel therapeutics with unique mechanisms of action. One particularly nefarious host is *E. coli* O157:H7, an enteric pathogen linked to a number of highly-publicized food recalls and whose enterohemorrhagic Shiga-like (Stx) toxins can cause kidney failure (hemolytic uremic syndrome) and even death (Eppinger et al., 2011). As a Jekyll-and-Hyde pathogen, *E. coli* O157:H7 gains much of its virulence and toxicity from Stx-encoding bacteriophages (Smith et al., 2007).

Because *stx* is largely silent in the lysogenic state, significant toxin production (and harm to the patient) does not occur unless the lysogen's Stx prophage(s) is/are induced (McGannon et al., 2010). Therefore, a strategy to prevent, disrupt, or otherwise disable Stx prophage induction would represent an innovative approach to addressing an important medical problem, one for which conventional antibiotic therapy is not always adequate or even advisable. Unlike most other bacterial infections which can be aggressively combatted with antibiotics, *E. coli* 0157:H7 infections pose a special challenge because the use of certain antibiotics can cause prophage induction, increased toxin production, and further harm to the patient (Zhang et al., 2000). This dynamic can be viewed as a biomedical hostage situation in which a terrorist (*E. coli* O157:H7) is holding a number of hostages (human cells) and has a dead man's switch (inducible Stx prophages) as a defense against the weapons of law enforcement (antibiotics). As with real-world hostage situations, the special medical challenge is to neutralize the microbial terrorist without triggering the dead man's switch and blowing up the hostages. One way to do this would be to disable the dead man's switch: having lost the ability to kill the hostages, the terrorist would no longer pose a significant threat and could be easily neutralized. The fundamental purpose of an anti-induction strategy, then, would be to eliminate *E. coli*'s ability to harm human cells by preventing the activation of its toxin-producing genes. Even though this anti-induction strategy would not be directly bactericidal, it might, by reducing bacterial toxin production, lessen the risk of serious complications, such as hemolytic uremic syndrome, that can result from *E. coli* O157:H7 infection. By defusing enterohemorrhagic *E. coli*'s molecular dead man's switch, a sufficiently effective anti-induction strategy might even enable traditional antibiotic therapy of *E. coli* O157:H7 infections: without the specter of prophage induction and toxin production, there would be little reason to avoid the use of antibiotics to combat these infections.

Thomas Edison famously said that genius is 1% inspiration and 99% perspiration, and indeed, the most difficult part of any applied science is not in the conceiving of new ideas but in their implementation. How, then, might an anti-induction strategy work in practice? One approach would be to utilize specific molecules that reduce prophage induction by eliminating *in vivo* inducing agents. Some of the most significant of these inducing agents are reactive oxygen species (ROS), such as hydrogen peroxide, that are produced as part of the innate immune response (Wagner et al., 2001). As powerful oxidizing agents, ROS decompose into free radicals, which can cause DNA damage to bacteria and, consequently, prophage induction. Therefore, one possible anti-induction strategy might involve molecules, such as the antioxidant glutathione (Liu et al., 2005), that scavenge free radicals and thereby decrease prophage induction. Other ROS scavengers, such as the manganese complexes found in *Deinococcus radiodurans* (Daly et al., 2010), might possess similar medical relevance.

Nevertheless, the molecular approach to stopping prophage induction has its limitations, even on a theoretical level. For one, the natural balance between antioxidants and pro-oxidants is a fine one, and because ROS and free radicals play important roles not just within the innate immune system but also in cell signaling and other normal physiologies (Townsend et al., 2003), it might prove detrimental to intentionally disrupt that balance. More importantly, the human body contains a plethora of chemically-distinct inducing agents, meaning that highly specific molecular approaches designed to bind or inhibit individual inducing agents (e.g., receptor mimics) would probably be insufficiently comprehensive. To have any significant therapeutic value, any anti-induction molecule would need to prevent induction by many different agents.

Another anti-induction strategy, based on basic phage biology and fundamentally different from any molecular-based approach, can also be envisioned. During normal conditions in *E. coli*, prophages are largely stable in lysogeny because the genes required for lytic growth are repressed by the repressor protein CI (Svenningsen et al., 2005). Because prophage induction only occurs when the concentration of repressor becomes too low (Ptashne, 2006), it would seem logical to predict that more repressor means less induction. Indeed, significant overexpression of CI repressor has been shown to strongly reduce prophage induction in *E. coli* (Czyz et al., 2001). Further, the carriage of multiple prophages by an *E. coli* lysogen has been shown to decrease prophage induction and Stx production (Serra-Moreno et al., 2008). If the presence of multiple prophages decreases induction, and if repressor overproduction further decreases induction, then *in vivo* overexpression of repressor by temperate phage co-infection could represent an alternate anti-induction strategy. For *E. coli* O157:H7, this might be achieved through the use of temperate coliphages, heteroimmune to Stx-encoding prophages, that have been genetically modified to lack a lytic cassette and to overexpress CI repressor. Importantly, this anti-induction strategy would be both corrective (by preventing prophage induction in Stx lysogens) and protective (by preventing commensal *E. coli*, not yet infected by Stx phages, from becoming toxinogenic during the latter stages of infection). By lacking the genes necessary for the lytic cycle, these modified phages would not propagate themselves beyond the initial infection and should not significantly impact gut microbiota, but as viral factories producing large quantities of repressor, they might be able to greatly reduce Stx prophage induction, Shiga toxin production, and enterohemorrhagic *E. coli*'s threat to human health.

Needless to say, these approaches are (for now) theoretical and would require significant scientific validation, not to mention regulatory approval, but the fundamental idea of developing anti-induction strategies remains compelling because the prominent role of prophage induction in bacterial pathogenesis extends far beyond *E. coli* O157:H7 to include infections caused by bacteria as varied as *V. cholerae*, *C. diphtheriae*, *S. aureus*, and group A streptococci (Wagner and Waldor, 2002; Cao et al., 2012). This paradigm of pathogenesis presents us with a powerful opportunity: by specifically targeting prophages and other mobile genetic elements, we might be able to apply our knowledge of the evolution of bacterial virulence to develop outside-the-box treatments for the Jekyll-and-Hyde pathogens that are significant contributors to human disease.
